# Single-Cell and Spatial Transcriptomics Explore Purine Metabolism–Related Prognostic Risk Model and Tumor Immune Microenvironment Modulation in Ovarian Cancer

**DOI:** 10.1155/humu/5530325

**Published:** 2025-05-09

**Authors:** Yinglei Liu, Rui Geng, Songyun Zhao, Jinjin Yang, Jinhui Liu, Yanli Zheng, Weichun Tang

**Affiliations:** ^1^Department of Obstetrics and Gynecology, The Second Affiliated Hospital of Nantong University (First People's Hospital of Nantong City), Nantong, China; ^2^Suzhou Center for Disease Control and Prevention, Suzhou, China; ^3^Department of Neurosurgery, The Affiliated Wuxi People's Hospital of Nanjing Medical University, Wuxi, China; ^4^Medical School of Nantong University, Nantong, China; ^5^Department of Gynecology, The First Affiliated Hospital of Nanjing Medical University, Nanjing, China

**Keywords:** ovarian cancer, purine metabolism, single-cell RNA sequencing, spatial transcriptomics, tumor microenvironment

## Abstract

**Background:** Ovarian cancer (OC) ranks as the second leading cause of gynecological cancer–related deaths in women globally. Single-cell and spatial transcriptomics could precisely describe the heterogeneity of OC that affect the clinical treatment.

**Methods:** Single-cell sequencing and spatial transcriptomics information were from different public datasets. A pseudotime analysis of cellular developmental pathways, score single-cell gene sets, and cell activity ratings in each metabolic pathway were performed. A prognostic model was created using univariate regression analysis, LASSO, and multivariate regression analysis. Finally, the immune microenvironment and immunotherapeutic effects were analyzed for their association with purine metabolism activity. Finally, RT-qPCR was used to estimate the mRNA level of OC in cell lines.

**Results:** We observed a higher purine metabolism score by a signature of 12 purine metabolism–related gene in tumor cells. When compared with fibroblasts, epithelial cells with high scores displayed more intense TGF-*β* signaling pathway activity. Forty-four differentially expressed purine metabolism–related genes were identified to be substantially expressed in the tumor's core region and were closely linked to purine and pyrimidine metabolic activities. Low-risk population had higher immune infiltration level and immunotherapy results. The NME6+ epithelial cell high-expression group had a greater prognosis and showed a negative connection with the tumor immune dysfunction and exclusion score and cancer-associated fibroblast cell concentration.

**Conclusion:** Purine metabolism was a predictor for OC patients' prognosis. The presence of positive NME6 expression in epithelial cells emerges as a protective factor for OC patients, presenting a possible therapeutic target for personalized treatment.

## 1. Introduction

Projected to reach 371,000 cases and an estimated 254,000 mortality in 2035, ovarian cancer (OC) ranks as the second cause of gynecological cancer–related deaths in women globally [1]. OC's high relapse rate and resistance to therapy result in a mere 30% 5-year survival rate [2]. Its diverse subtypes, characterized by distinct biological and molecular features, create disparities in treatment accessibility. Despite the success of emerging immunotherapies targeting PD-1 and PD-L1 against various cancers, OC has shown limited response. This has prompted researchers to explore alternative therapeutic targets to enhance OC patients' prognoses.

Identifying connections between cell types is vital for understanding the prognosis and treatment of tumors. scRNA-seq technologies offer crucial insights into gene expression at single-cell level [3]. This enables the exploration of cellular components and their interactions within tumor microenvironments, even unveiling novel cell types with diverse functions [4]. However, the process of sequencing a single cell inevitably results in the loss of location information, impeding the assessment of spatial heterogeneity across tissues at the single-cell transcriptome level. Recent advancements in spatial transcriptomic technologies have opened exciting opportunities to simultaneously capture spatial location information and gene expression data of cells, thereby yielding novel insights across diverse research domains. Nonetheless, the comprehensive exploration of tumor microenvironment and immunological phenotype of OC at the single-cell resolution remains an ongoing challenge.

A key characteristic of cancer is unrestrained cell growth [5]. Purines play a pivotal role as primary sources of intermediates in the metabolic processes of cancer cells and are essential components of nucleotides required for cell growth [6]. Scientific speculation suggests the presence of specialized metabolic compartments in the body, such as the purinosome for purine metabolism, housing the requisite enzymes for this metabolic pathway [7]. This arrangement enables cancer cells to effectively synthesize purine nucleotides through the de novo synthesis pathway. Despite its potential in assessing cancer subtype [8, 9], the significance of purine metabolism in OC has received limited attention.

Therefore, by incorporating spatial information into the analysis to show the mutational landscape of OC subtypes, we constructed a series of tissue-specific clusters using scRNA-seq and spatial transcriptomic data. These clusters provide a thorough and understandable mutational landscape of gene expression in OC tissues. The mechanism of purine metabolism genes in promoting the progression of OC was examined using bulk RNA-seq from the TCGA. A validated prognostic model was created on the basis of this information and assessed for the degree of immune infiltration and immunotherapy effectiveness. As a possible immunotherapy target, NME6 was discovered. This will generate fresh concepts for the creation of novel OC treatments.

## 2. Materials and Methods

### 2.1. Datasets

Bulk RNA-seq data, mutation data, and clinical characteristics of TCGA-OV were retrieved from the UCSC Xena website. There were 360 samples that contained survival information, and the expression profiling data were in TPM format. The validation model used gene expression profiling information of 278 OC patients from GSE9891. The “limma” and “sva” software tools were applied to combine the two data and correct for batch effect [10]. TISCH gathered 76 sets of tumor single-cell transcriptome data from the Gene Expression Omnibus (GEO) database and ArrayExpress together with associated patient data. The data spanned 27 different cancer types. The EMTAB8107 cohort of TISCH, which has a total of 24,781 cells from cancer and paracancerous tissue, is where the OC single-cell sequencing data came from [11]. From GSE203612, the primary OC spatial transcriptome data for the 10x Visium platform was retrieved [12]. The term “purine metabolism” was used to derive 163 purine metabolism–related genes (PMRGs) from the Molecular Signatures Database (MSigDB) (Table S1).

### 2.2. Analysis With Single-Cell RNA Sequencing Data

The scRNA-seq data were analyzed using the “Seurat” and “SingleR” R packages [13]. We only retained genes present in more than two cells and filtered out cells with less than 200 or more than 10,000 genes, less than 1000 molecules, and more than 20% of nuclear and translational genes. Normalization was conducted for the data using “NormalizeData” from the R package “Seurat.” With the “FindVariableFeatures” function, the first 2000 highly variable genes were identified. After that, principal component analysis (PCA) was applied to minimize the dimensionality of the data. Essential principal components (PCs) were determined using the JackStraw analysis, with the top 20 PCs selected. For cell clustering, we utilized the “FindNeighbors” and “FindClusters” functions on the integrated data and visualized cell clusters by UMAP and tSNE. Marker genes for various cell types were identified through a Wilcoxon test, carried out using the “FindAllMarkers” and “FindMarkers” routines. We annotated different cell subpopulations by merging data from the original text with annotations from the TISCH database, relying on common marker genes.

Package “Monocle” was applied for the pseudotemporal analysis of cellular developmental trajectories in epithelial cells [14], and unsupervised analysis was utilized to choose developmental differential genes. Differential gene testing for the pseudotime analysis was based on a cutoff for significance *q*-value < 0.01. DDRTree was used to reduce the dimensionality of the data. We scored the gene set for single-cell data using five widely used techniques, including AddModuleScore, single-sample Gene Set Enrichment Analysis (ssGSEA), AUCell, UCell, and singscore, based on the expression of 163 PMRGs. With the “AddModuleScore,” we computed the average expression value for each gene, divided the expression matrix into segments accordingly, and randomly selected control genes as background values from each segment [15]. “AUCell” employed the area under the curve to determine whether an input gene collection is enriched for expressed genes in the samples [16]. Based on the gene expression patterns in single-cell transcriptome data, we clustered cells using techniques like dimensionality reduction and assigned a predicted cell type label to each cell [17]. A cell state evaluation technique called a “singscore” was used to measure how active a specific biological function or process is in a single sample or cell. Taking into account the weights and orientations of the genes, it determines the cell state score of a sample or cell on the basis of the set of genes in a gene expression profile.

Finally, the “CellCall” tool combined intracellular and intercellular data to infer intercellular communication networks [18]. The ability to combine intracellular transcriptional factor expression with intercellular ligand–receptor communication, which enables the analysis of changes in receptor cellular pathways brought on by the communication between two specific cell types, is its most significant feature.

### 2.3. Analysis With Spatial Transcriptomic Data

Additionally, Seurat in R was used to analyze spatial transcriptome data. The function “SCTransform” was used to normalize, scale, and identify the most variable features in UMI counts. After that, the UMI counts were downscaled, and unsupervised cluster analysis was performed using “RunPCA.” Subgroups and genes were visualized using the SpatialFeaturePlot tool. The “scMetabolism” R package is in view of a standard single-cell matrix file and scores each cell using the VISION algorithm to determine how active the cell is in each metabolic pathway [19].

Scanpy is a Python-based software suite that includes preprocessing, visualization, clustering, and differential expression analysis. The University of Queensland's Institute of Molecular Biosciences produced the stlearn software, which was used to combine data from ligand pairings and gene expression to infer relationships.

Reverse Compositional Transcriptomics Deconvolution (RCTD) calculates the geographic distribution and relative abundance of cell types [20]. Multiview Intercellular SpaTial Modelling Framework is an interpretable multiview framework based on machine learning for parsing intercellular interactions in highly multiplexed data. It allows researchers to analyze spatial transcriptomic data without the necessity for cell type identification, and it offers fresh insights and hypotheses that help in the understanding of cellular interaction patterns and mechanisms [21]. MISTy is now available as a R package named mistyR, complete with full documentation and instructions.

### 2.4. Construction of Prognostic Models

The training set was the TCGA-OV cohort, and the validation set was the GSE9891 dataset. First, univariate cox analysis was utilized to find genes statistically correlated with overall survival (OS) using 163 PMRGs. Following that, we utilized LASSO and multivariate regression to look for factors that were closely connected with prognosis. A risk score was computed as follows:

 $$\begin{array}{c} \text{Riskscore}=h0\left(t\right)*\exp\left(b1*x1+b2*x2+\cdots +\text{bp}*\text{xp}\right), \end{array}$$

where *h*0(*t*) is the baseline risk, denoting the risk when all the independent variables are 0; *b*1, *b*2, ⋯, bp represent the regression coefficients; and *x*1, *x*2, ⋯, xp represent the independent variables, which are observations at time *t*. All the participants were divided into two scoring groups based on the median value of their scores.

### 2.5. Analysis of Nomograms and Mutations

We built a nomogram to visualize the chance of survival. We inferred the net advantage of the nomogram and clinical features alone using consistency index analysis and decision curve analysis (DCA). The R “oncoplot” function was applied to investigate detailed mutation profiles.

### 2.6. The Evaluation of Immunotherapy and Immunological Microenvironment

The “estimate” was applied to assess malignant tissue mesenchymal and immune cell abundance [22]. The amount of immune infiltration was then evaluated using the TIMER 2.0, which incorporates the outcomes of seven assessment approaches. In the form of a heat map, these data were used to quantify the relative fraction of immune cell infiltration. ssGSEA can assess the proportions of immunological-related activities. We assessed the two gene sets by the GSVA technique to analyze the connection with risk scores. The association between selected genes and immune genes was explored further [23]. GSEA was carried out comparing two groups through the MSigDB database “c2.cp.kegg.v6.2.symbols.gmts” file in order to explore the differences in biological functions between various populations. Tumor immune dysfunction and exclusion (TIDE) stands for tumor immune dysfunction and rejection. It is a computational framework for analyzing the gene expression profiles of tumor samples to determine the possibility of tumor immune escape. Comprehensive immunogenomic analysis results are conducted via the Cancer Immunome Atlas (TCIA) web application. Immunophenotype score (IPS) ranges from 0 to 10 [24].

### 2.7. qRT-PCR to Verify the Expression of NME6 in Samples

Three families of ovarian cells were obtained. The acquisition process and sample storage and processing are the same as before [25]. The extraction of RNA and the implementation of qRT-PCR were also operated strictly in accordance with the protocols. The forward sequence primer is TCCAGCTCACTCTAGCCCTG, and the reverse sequence primer is CGGTAAAACCTCTGGCAATCTT.

### 2.8. Statistical Analysis

R 4.1.3 and Python 3.9 were used to conduct all of the analyses. The Kaplan–Meier survival analyses and log-rank tests were applied to each dataset in order to assess predict values and compare the survival of patients in various subgroups. The connection of continuous variables was examined by the nonparametric Wilcoxon rank sum test. *p* < 0.05 was regarded as statistically significant.

## 3. Results

### 3.1. Single-Cell Enrichment Scores for Genes Involved in Purine Metabolism

**Figure 1:**
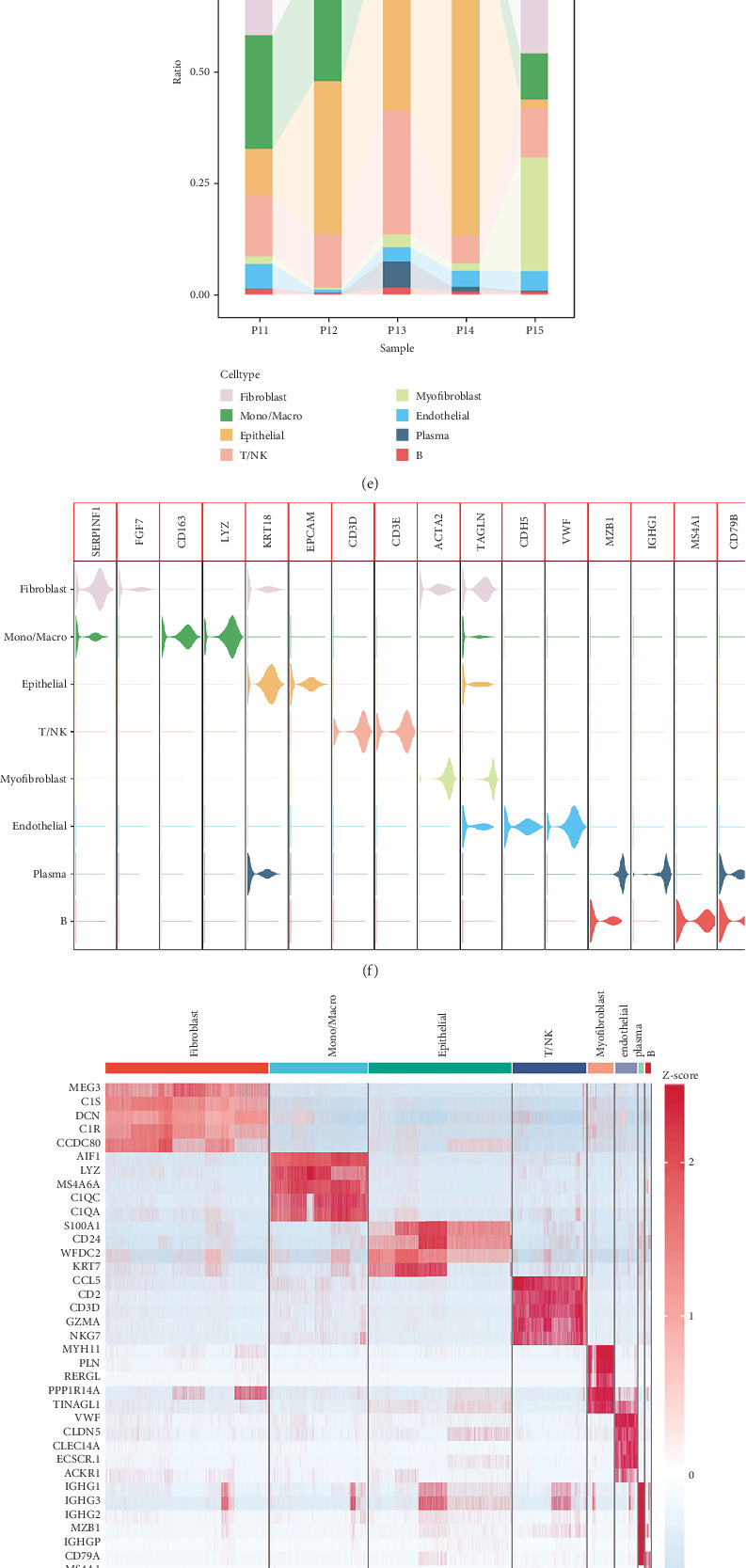
Classification of OC's purine metabolism–related gene enrichment scores and cell subpopulations. (a–d) tSNE plots of different samples, tissues, clusters, and cell subgroups. (e) A histogram showing the composition of several samples' cell types. (f) The expression of common genes in these cell subpopulations that are used for cellular annotation. (g) The relative expression of marker genes. Gene expression levels are represented by red and blue, respectively. (h) Bubble plots were used to show the purine metabolism–related gene enrichment scores for each kind of OC cell. (i) UMAP plots show the enrichment scores of genes involved in purine metabolism for each cell type, with deeper green scores indicating greater enrichment scores. (j) Differences in the enrichment scores of PMRGs in each cell types. ns, no significance; ⁣^∗^*p* < 0.05; ⁣^∗∗∗∗^*p* < 0.0001.

In order to explore the heterogeneity of OC and to evaluate variations between cells within and near the tumor, we analyzed cell types and gene expression within them. The PCA was used to perform dimensionality reduction using 2000 highly variable genes, and the top 20 PCs were selected for additional analysis. Following the identification of 33 distinct cell clusters, we integrated cell annotations from the original literature using established marker genes to annotate these clusters (Figures 1a, 1b, 1c, and 1d). The percentage of various cell types in the five samples is shown in Figure 1e. Using several well-known cell marker genes, we demonstrated the expression of these genes in each type of cell (Figure 1f). Additionally, a heat map displays the relative expression of the estimated marker gene in each cell subpopulation (Figure 1g). We employed the five most popular methods to score the gene sets based on the expression of 163 PMRGs. Purine metabolism scores (PMSs) were comparatively greater in epithelial or tumor cells, as seen in Figure 1h,i. We also found that the PMSs were comparatively higher in tumor-associated monocyte macrophages, fibroblasts, T/NK cells, and epithelial cells (Figure 1j). We identified 44 genes correlated with purine metabolism between epithelial cells in tumors and normal tissues (Table S2).

### 3.2. Pseudotime Analysis and Intercellular Communication Analysis

**Figure 2:**
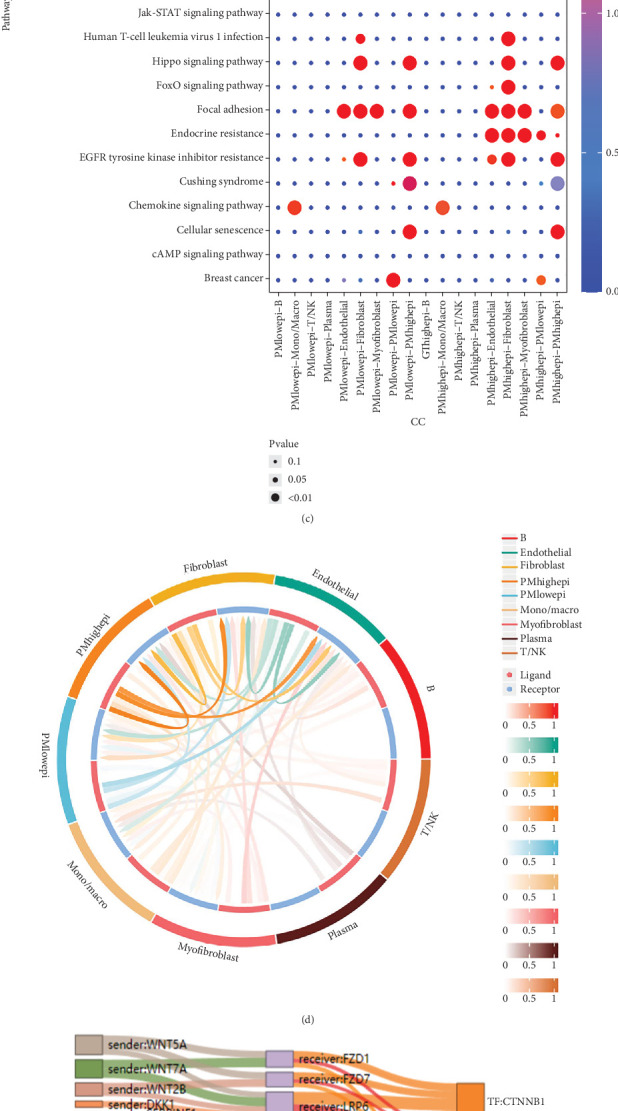
Analyses of the cell communication and developmental trajectories. (a) Trajectory inference and pseudotime analysis. (b) Heat map displaying the expression of 44 genes associated with purine metabolism that are differently expressed during cell development. (c) Activity analysis of signaling pathways is shown in a bubble diagram. (d) A circle diagram illustrating the differences in ligand receptor strength between various cell types. (e) Ligand–receptor pairs and transcription factors between fibroblasts and epithelial cells. (f) Differences in ligand receptor strength between each type of cells.

In total, we counted three distinct cell states in developing epithelial cells. Cluster 4 is an illustration of the early stage of cell formation (Figure 2a). The heat map in Figure 2b shows the expression of 44 PMRGs that are differently expressed at various developmental stages.  Figure 2c displays the analysis results for the investigation of signaling pathway activity. For instance, fibroblasts displayed higher levels of TGF-*β* signaling pathway activity in epithelial cells with high scores. The TGF-*β* signaling pathway is essential for the growth and development of cells and tissues. It also has a vital regulatory correlation with the regulation of cell proliferation, interstitial cell production, differentiation, and death, as well as immune function, inflammatory response, organ formation, and the healing of wounds. The loops in Figure 2d show the strength of ligand receptor signaling between different cell types, with high-scoring epithelial cells exhibiting stronger cellular interactions with fibroblasts. High-scoring epithelial cells interacted with fibroblasts through ligand–receptor pairs such as WNT7A-FZD1 more strongly (Figure 2f). We then focused on ligand–receptor pairs and associated transcription factors between epithelial cells and fibroblasts with higher PMS (Figure 2e).

### 3.3. Purine Metabolism–Related Characteristics in stRNA-Seq

**Figure 3:**
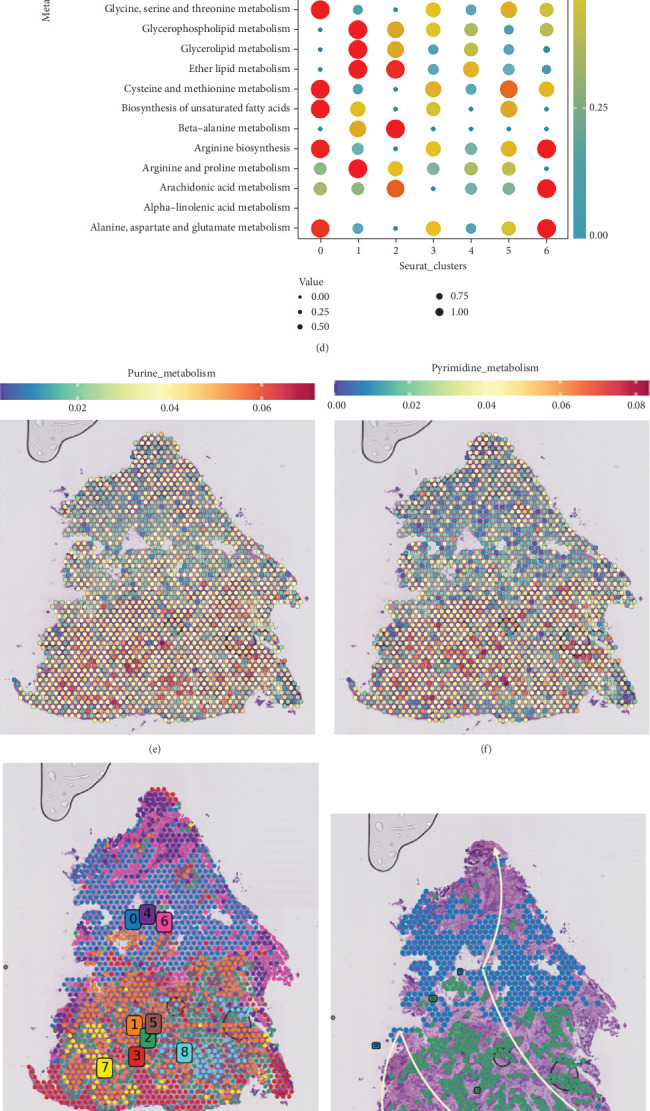
Correlation analysis of spatial transcriptomes. (a) The seven clusters discovered by stRNA-seq are shown in the UMAP plot. (b) Spatial map displaying the seven clusters discovered by stRNA-seq. (c) Expression levels of PMRGs in different clusters are shown in the bubble plot. (d) Bubble plots showing the metabolic activity of several clusters. (e) Spatial map of purine metabolic intensity. (f) Spatial plot of pyrimidine metabolic intensity. (g) Spatial map of the nine cell clusters. (h) Spatial map depicting the developmental pattern of Cell Clusters 0 to 2. (i) Spatial distribution of various cell types. (j) Spatial clustering of various cell types inferred from MISTy results. (k) Spatial correlation of various cell types inferred from MISTy results.

A total of seven cellular subpopulations were obtained in space after downscaling and clustering the spatial transcriptome sequencing data of an OC patient. The SCTransform's method was used to correct the sequencing depth and a series of normalization processes (Figure 3a,b). Subpopulation 0 was predominately found in the tumor core of the OC, as indicated by annotations in the original literature, and 44 differentially expressed PMRGs in the prior study were strongly expressed in Subpopulation 0 (Figure 3c). Among the metabolic activity of several cell subpopulations, Subpopulation 0 was intimately linked to metabolic processes involving purines and pyrimidines (Figure 3d). Purine and pyrimidine metabolic activities were primarily abundant in the tumor's core, as seen in Figure 3e,f. After normalizing and clustering the spatial transcriptome data, we obtained nine cell subpopulations (Figure 3g). It is interesting to note that Cluster 0, which is located in the peripheral region of the tumor, differentiates toward Cluster 2, which is located in the tumor's core, to carry out cell differentiation (Figure 3h). Finally, we deduced the dominating cell type at each position in the space. The core part of the tumor was where epithelial cells with low and high PMSs were concentrated, whereas the tumor's periphery was where fibroblasts were concentrated (Figure 3i). The MISTy results revealed that epithelial cells with higher PMS interacted with fibroblasts more frequently in space (Figure 3j,k).

### 3.4. Building and Evaluating Prognostic Models

**Figure 4:**
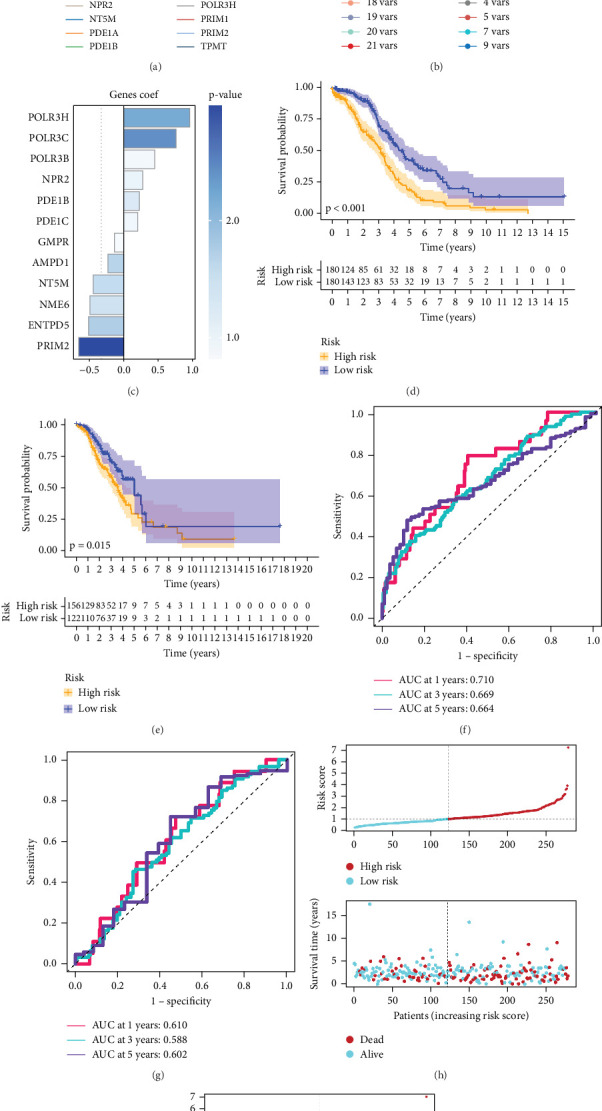
Calculation and prognostic modeling of risk scores associated with purine metabolism. (a) LASSO coefficient curves. (b) 10-fold crossvalidation used to adjust parameter selection. (c) Identified model genes and coefficients. (d, e) Survival curves of patients in each group. Risk score AUC values for the (f) TCGA cohort and the (g) GSE9891 cohort. Risk distribution and patient survival in the (h) TCGA cohort and the (i) GSE9891 cohort.

One hundred and sixty-three PMRGs were applied to build predictive signatures in bulk-seq for OC in order to assist clinical decision-making. Three hundred and sixty OC samples from the TCGA and 278 OC samples from the GSE9891 were used as the training set and the external validation set, respectively. Firstly, 26 PMRGs affecting the OS of OC patients were screened out (Table S3). A 12-gene signature was created to prevent overfitting of prognostic characteristics and to focus the genes that predict OS (Figure 4a,b). Based on the formula, risk ratings were computed for each patient (Figure 4c). Our model was utilized to determine the risk score using the previously mentioned formula (Table S4). For each patient, risk maps display specific survival results (Figure 4h,i). Participants with high risk had lower OS compared to those in the low-risk group, according to the survival curves (Figure 4d,e). It also showed great performance in inferring OS, according to the ROC curves (Figure 4f,g).

### 3.5. Nomograms and Clinical Features

**Figure 5:**
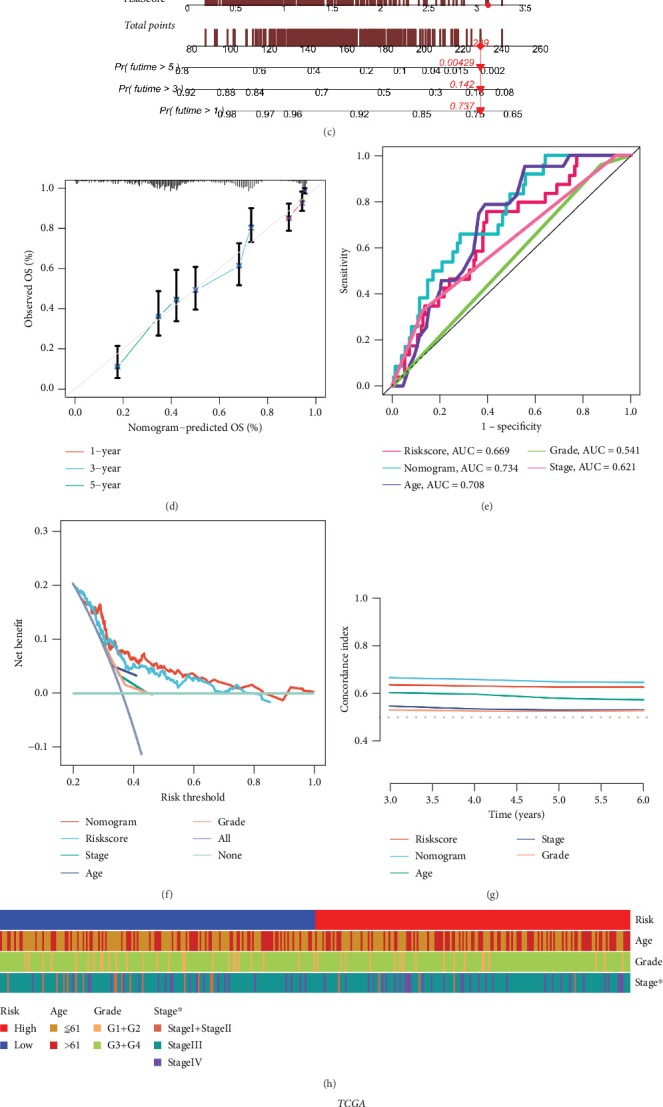
Independent prognostic assessments of the TCGA cohort's risk score and clinical variables. (a, b) The result of univariate and multivariate Cox regression analysis. (c, d) The nomogram and correction curves. (e) One-year AUC curves for risk score and other single characteristics. (f) DCA curves for risk score, nomogram score, and other clinical variables. (g) Using c-exponential curves, to assess the predictive performance of different variables. (h) Chi-square test heat map of clinical features related with subgroups. (i) Proportion of stage in the groups.

The risk score is an independent predict factor when compared to other features (age, grade, and stage) (Figure 5a,b). In view of previously mentioned link between clinical characteristics and risk ratings, we constructed a nomogram to predict the OS (Figure 5c). The calibration curves also demonstrated that it could generate accurate predictions (Figure 5d). Furthermore, the risk score and nomogram score had a substantially higher AUC at 3 years than other clinicopathological variables (Figure 5e). The 3-year DCA curves and *C*-index revealed that our risk score had the biggest net benefit (Figure 5f,g), and risk grouping was connected with patient STAGE (Figure 5h). The high-risk group was classified at a higher level (Figure 5i). We are more certain that the risk score and nomogram are a reliable clinical prediction scoring system based on the outcome analysis.

### 3.6. Mutational Landscape

**Figure 6:**
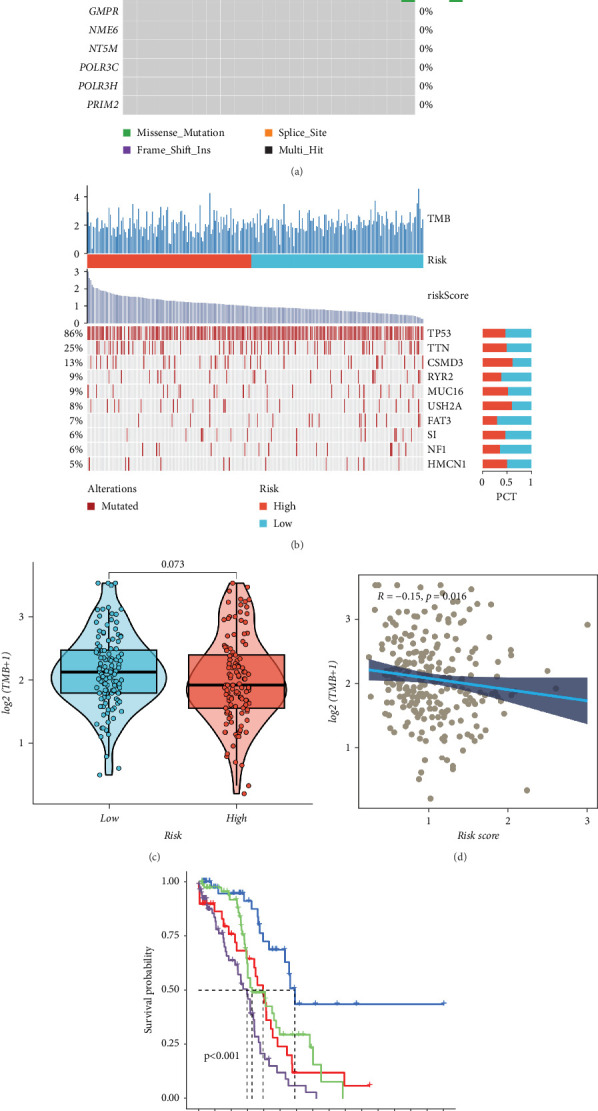
Landscape of variation in OC. (a) The mutation landscape for each of the 12 PMRGs. (b) The top ten genes' mutation landscape in terms of mutation frequency in the two risk score categories. (c) TMB comparison between groups. (d) A correlation analysis of the risk score and the TMB. (e) Survival differences between the subgroups.

Given that genetic mutations have great association with a patients' personalized treatment. The most often mutated type of these genes, as indicated in Figure 6a, was missense-mutation. However, the overall mutation frequency was exceedingly low. The distribution of the most often altered genes in OC in risk score groupings was studied (Figure 6b). TMB, however, did not differ across individuals in the groups (Figure 6c,d). The patients were then divided into four groups in view of their median TMB values and risk scores. The results revealed that patients with low risk scores and high mutations had a relatively better prognosis (Figure 6e).

### 3.7. Analysis of Immune Infiltration and Immune Function

**Figure 7:**
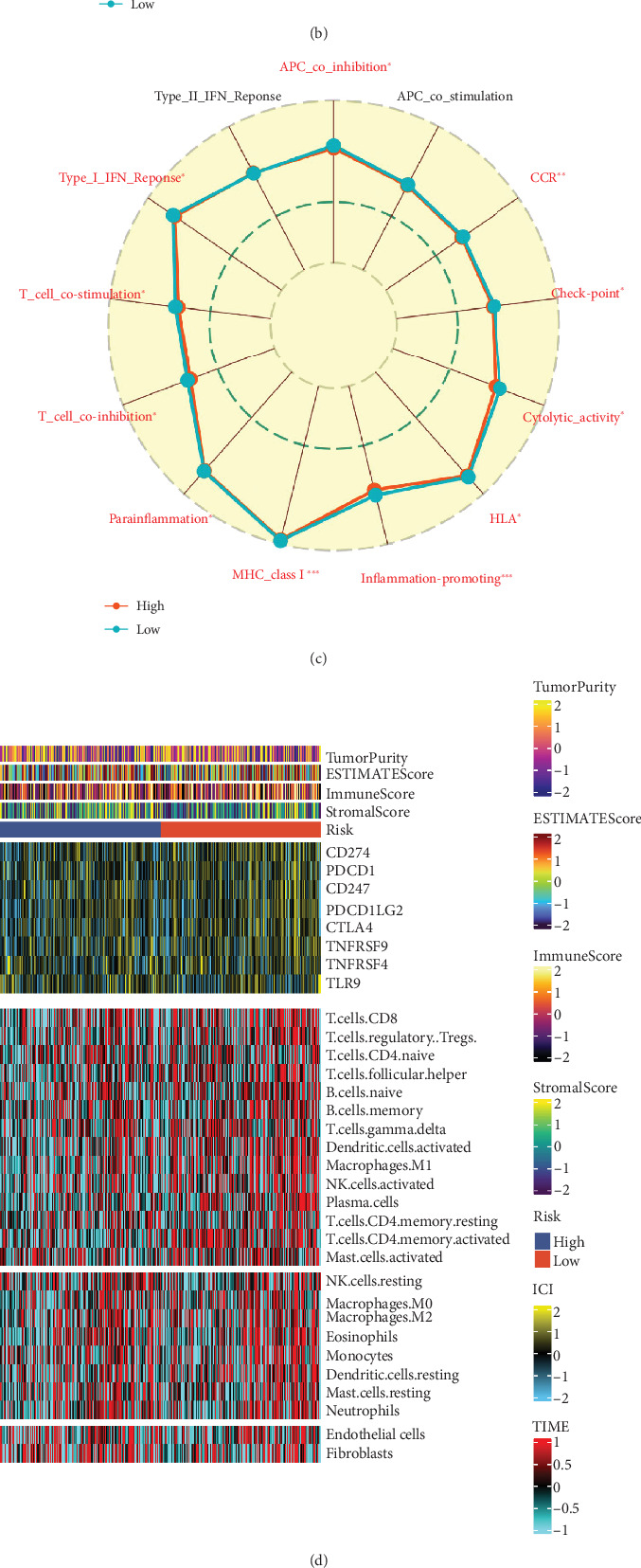
Analysis of immune microenvironment. (a) Seven methods were utilized to evaluate the differences in immune infiltration in the subgroups. (b, c) Radar plots showing differences of patients with different risk categories as determined by ssGSEA. (d) Heat map showing changes in immune checkpoint expression, immune cell infiltration, and TME score (based on ssGSEA) in different risk categories. (e) Changes in immune checkpoint expression in the subgroups. (f, g) GSEA of scoring subgroups.

Clinical results and treatment response of patients are influenced by the TME. Among them, tumor-infiltrating immune cells (TIICs) have a substantial effect on the advance of tumors and the effectiveness of antitumor treatments. TIIC's distribution and composition are directly connected to the tumor development [26]. Therefore, we evaluated the immune landscape of the high- and low-risk score groups (Figure 7a). According to the findings, immune cell infiltration and immune pathway scores were higher in low-risk groupings (Figure 7b,c). Nearly all ICs were more expressed in the grouping with low risk scores (Figure 7e). Additionally, the immune complexes (ICs), immunological scores, and immune cell infiltration in various risk categories were shown using heat maps (Figure 7d). We used GSEA to investigate any potential changes in biological function and chose the four most crucial signaling pathways for each due to the differing prognoses and immune infiltration profiles of patients in subgroups (Figure 7f,g). The low-risk subgroups were primarily enriched in immune response and cytokine pathways, whereas the high-risk subgroups were primarily linked to signaling pathways in various types of tumors. The immunosuppressive microenvironment of OC tumors may correspond to patients with higher risk scores, which could lead to a decreased incidence of immunotherapy response.

### 3.8. Prediction of the Outcomes of Immunotherapy

**Figure 8:**
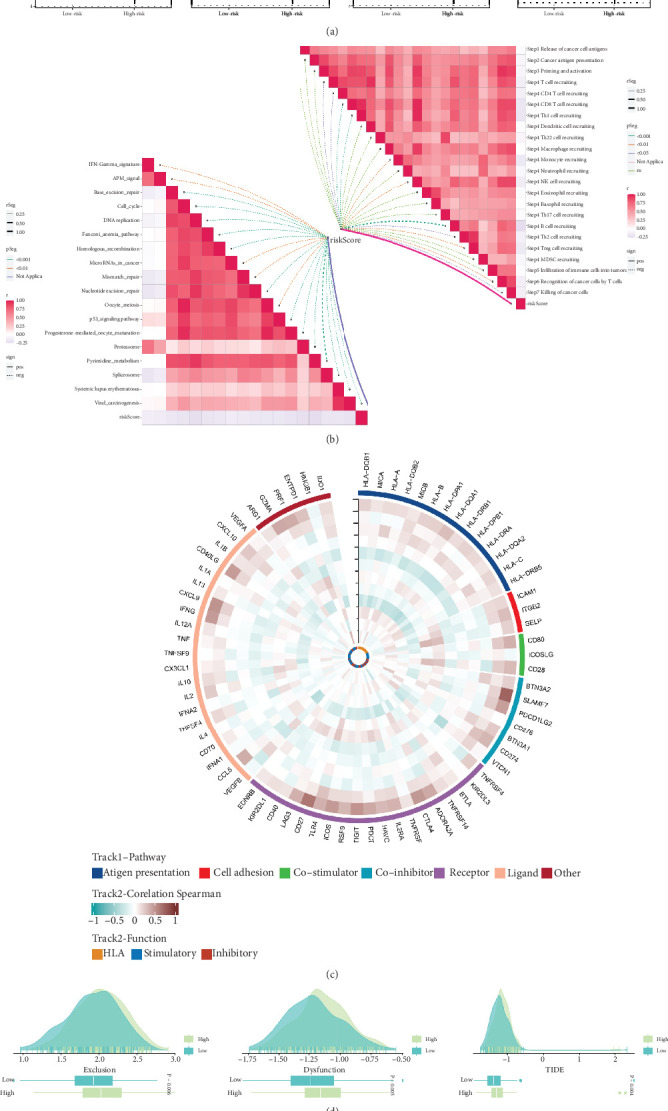
Immunotherapy result prediction. (a) A comparison of the IPS distribution between the subgroups. (b) Associations between the risk score, the ICB response, and the stage of tumor-immunity cycle. (c) Gene-immunity gene correlations heat map. (d) TIDE between the groups of OC patients. ⁣^∗^*p* < 0.05; ⁣^∗∗^*p* < 0.01; ⁣^∗∗∗^*p* < 0.001; ⁣^∗∗∗∗^*p* < 0.0001.

With greater IPSs indicating stronger responses to ICB, violin plots demonstrate the link between risk groups and IPSs. Individuals from the low-risk group were thought to respond favorably to immune checkpoint inhibitors (Figure 8a). We then examined the associations between risk scores and ICB response profiles because the immune microenvironment mediates ICB responses and discovered that there was a substantial inverse relationship between risk scores and these ICB response profiles (Figure 8b). We conducted correlation analyses utilizing 12 model genes and traditional immune-related genes to further explore the variations in immune responses between groups (Figure 8c). The gene expression profile of tumor samples is reflected by TIDE as a measure of the likelihood of tumor immune escape. Poorer ICB response and patient survival were linked to higher tumor TIDE prediction scores. Above all, the high-risk group exhibited greater dysfunction and exclusion scores as well as TIDE scores that were relatively higher (Figure 8d).

### 3.9. NME6+ Epithelial Cells as a Potential Immunotherapy Target

**Figure 9:**
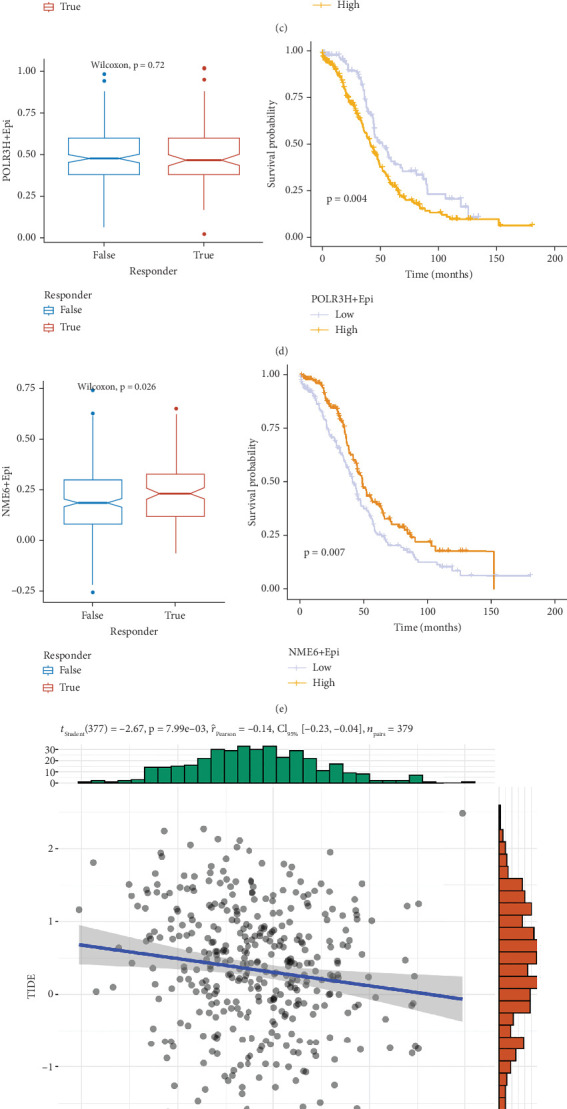
NME6 is a protective variable of OC. (a) Three genes were founded by using differential analysis in scRNA-seq. (b) A spatial map of GMPR, NME6, and POLR3H expression in OC. (c) OS curves for patients with GMPR+ epithelial expression. TIDE predicted immunotherapy response for high and low percentages of GMPR+ epithelial cells. (d) Survival curve of patients with POLR3H+ epithelial cell expression. According to TIDE, high and low percentages of POLR3H+ epithelial cells indicated immunotherapy response. (e) Survival curves of patients with NME6+ epithelial cell expression. According to TIDE, high and low percentages of NME6+ epithelial cells indicated immunotherapy response. (f, g) Correlation of NME6+ epithelial cell content with TIDE score and cancer-associated fibroblast cell content.

According to the intersection of the genes with the 44 model genes in Table S2, three PMRGs were considered to affect the progression and treatment of OC (Figure 9a). The spatial map shows the expression of GMPR, NME6, and POLR3H (Figure 9b). We categorized single-cell sequenced epithelial cells into the expression-positive and expression-negative groups. There was no significant difference between the TIDE results and survival analysis results for GMPR+ and POLR3H+ epithelial cells (Figure 9c,d). The high NME6+ epithelial cell expression group had a better prognosis and statistically significant difference in the TIDE scores (Figure 9e). The number of NME6+ epithelial cells showed significant differences in the TIDE scores and the amount of cancer-associated fibroblast content, and they were all significantly negatively correlated (Figure 9f,g). Therefore, we speculated that NME6-expressing positive epithelial cells would be a protective factor for OC patients. We also confirm the expression of NME6 in IOSE and OVCAR3 which are normal ovarian epithelial cell lines and OC cell lines, respectively (Figure S1, Table S5). What can we see is that normal ovarian cell lines have higher NME6 levels than those in OC cell lines.

## 4. Discussion

Purines promote tumor cell proliferation by generating nucleotides via the de novo and salvage pathways [27], whereas purines operate as potent modulators of immune cell responses and have been demonstrated to have a significant impact on immune cell signaling [28, 29]. Purines also offer the energy and cofactors required for cell survival and growth [30]. All in all, purines are strongly associated to cancer progression [31, 32], and the utilization of purine metabolism for tumor suppression offers promising future research possibilities.

In this study, PMS was higher in tumor cells, particularly epithelial cells. The great majority of ovarian malignancies are epithelial tissue ovarian tumors [33]. The ovarian surface epithelium is thought to be less differentiated than other genital tract epithelia and invaginates into the underlying stroma, leading to inclusion cysts and eventually malignant transformation [34]. Through self-renewal and the ability to generate more specialized, differentiated cell types, epithelial stem cells provide crucial physiological activities in the formation, maintenance, and repair of numerous tissues. Given that epithelial cells are the most commonly dysregulated cell type in OC, epithelial cells' high PMS suggests that purine metabolism within epithelial cells may be connected with OC progression. Spatial transcriptome analysis results revealed a close relationship between the purine metabolism activity center and the core subpopulation, confirming the role of purine metabolism in OC progression once more.

Epithelial cells with high PMS have shown robust cellular contact with fibroblasts in OC tissues. Fibroblasts originate from the mesenchyme of the embryonic mesoderm and are also believed to be crucial in the genesis of tumors. Epithelial mesenchymal transition (EMT) is a process whereby epithelial cells can also give rise to fibroblasts [35]. EMT is believed to increase cancer cell invasiveness and circulating tumor cells and encourage medication resistance [36]. TGF-*β* regulates a number of physiological processes, including cell proliferation, differentiation, control of cell death, embryonic development, immunological function, and inflammatory response [37]. TGF-*β* is one of the key transduction mechanisms governing EMT. TGF-*β*, in particular, inhibits effector T cells and stimulates tumor-associated macrophages and immunosuppressive T regulatory cells, which helps cancer escape and prevents the exhaustion of the immune system's capacity to respond [38]. The tumor is “cool” at this point because it does not cause an immunological reaction. In breast, colorectal, and prostate cancer, increased TGF-*β* expression in the tumor area is linked to a poor prognosis and locally progressed disease [39]. Unsurprisingly, fibroblasts and endothelial cells in OC in our investigation displayed robust TGF-*β* signaling pathway activation. Therefore, we hypothesize that through increasing the TGF-*β* signaling pathway, epithelial cells and fibroblasts collaborate to produce EMT, which then advances OC.

Because OC exhibits cellular, molecular, and geographical heterogeneity, using the immune system to combat it is a viable strategy. Interventions such immune checkpoint inhibition, cancer vaccinations, and overt cellular treatments have been used in earlier trials [40]. Despite the fact that PD-L1/PD-1 has been identified as a target for immunotherapy and that these immunotherapies have demonstrated effectiveness in a number of malignancies, the efficacy of immunotherapy in OC is not sufficient [41–43]. The low-risk group has higher immunological activity, according to our risk assessment of patients using a predictive model of OC with 12 genes related to purine metabolism. Purine metabolites can exert protumourigenic effects through a variety of mechanisms, including direct toxicity, interference with the TME, promotion of DNA synthesis, and DNA damage repair. Patients with higher risk scores may correspond to a tumor-immunosuppressive microenvironment of OC and have a worse prognosis [44]. Helper T-cell immunological infiltration in ovarian TME, particularly TH1 cells, TH2 cells, and Tfh cells, was considerably higher in low-risk participants. By enhancing cellular and humoral immunity, higher T-cell subsets within the TME may increase patient survival in solid organ tumor types with nonlymphocyte origin, such as OC [45, 46]. In the high-risk groups, a suppressive immunological milieu may be a factor in the decreased immunotherapy response rates. IPS and TIDE can help identify people who potentially benefit from immunotherapy, that is, those with lower risk scores, by revealing information about the responsiveness of tumor cells to immunotherapy, which is crucial to determining efficacy and choice of therapeutic drugs.

An important finding of this study is that NME6, a monomeric, phosphotransfer-inactive member of the NME/NDPK family involved in nucleoside diphosphate phosphorylation as well as in its pathogenic, therapeutic, and developmental effects, may act as a protective factor in OC patients. Phosphorylation and several regulatory processes connected to metastasis, malignant transformation, and development [47]. The mechanism of action of this family member in different malignancies is yet unknown, despite the apparent crucial function of this protein family in many physiologic and pathological processes [48]. On the one hand, NME6 has the potential to contribute to signaling by boosting nucleoside metabolism [49]. On the other hand, NME6 can suppress p53-induced apoptosis [50]. Although we believe that NME6 is a protective factor for OC, NME6 has been shown to be a melanoma promoter [51]. Therefore, NME6 may act as an oncogene, and more studies are still needed.

In conclusion, our study used scRNA-seq and spatial transcriptome to examine single-cell and tissue spatial gene expression patterns in OC patients, revealing significant heterogeneity within individual tumors. NME6 was first suggested as a protective factor for OC and as a potential target for immunotherapy. However, there are certain restrictions on this study. In order to fully understand the intricacy of OC, more samples need to be analyzed than were included in this study. Second, even though we have discovered NME6 as a possible immunotherapeutic target in OC, more research needs to be done on its mode of action, and genuine trials need to be conducted to confirm its true efficacy.

## Data Availability

The datasets used and/or analysed during the current study are available from the corresponding author on reasonable request.

## Supporting Information

Supporting Information 1 Figure S1. The expression of NME6 in IOSE and OVCAR3 cell lines.

Supporting Information 2 Table S1. The list of 163 purine metabolism-related genes from the Molecular Signatures Database.

Supporting Information 3 Table S2. The list of 44 genes involved in purine metabolism expressed differently in tumors and normal tissues.

Supporting Information 4 Table S3. The list of 26 purine metabolism-related genes that affecting the overall survival (OS) of OC patients.

Supporting Information 5 Table S4. The variables and coefficients of the model assessed through cox regression.

Supporting Information 6 Table S5. Relative expression of NME6 in normal and tumor cell lines.

## References

[1] Barua A., Bahr J. M. (2022). Ovarian Cancer: Applications of Chickens to Humans. *Annual Review of Animal Biosciences*.

[2] Siegel R. L., Miller K. D., Fuchs H. E., Jemal A. (2021). Cancer Statistics, 2021. *CA: A Cancer Journal for Clinicians*.

[3] Sun Y., Wu L., Zhong Y. (2021). Single-Cell Landscape of the Ecosystem in Early-Relapse Hepatocellular Carcinoma. *Cell*.

[4] Dai H., Li L., Zeng T., Chen L. (2019). Cell-Specific Network Constructed by Single-Cell RNA Sequencing Data. *Nucleic Acids Research*.

[5] Yin J., Ren W., Huang X., Deng J., Li T., Yin Y. (2018). Potential Mechanisms Connecting Purine Metabolism and Cancer Therapy. *Frontiers in Immunology*.

[6] Ma F., Zhu Y., Liu X. (2019). Dual-Specificity Tyrosine Phosphorylation-Regulated Kinase 3 Loss Activates Purine Metabolism and Promotes Hepatocellular Carcinoma Progression. *Hepatology*.

[7] An S., Kumar R., Sheets E. D., Benkovic S. J. (2008). Reversible Compartmentalization of De Novo Purine Biosynthetic Complexes in Living Cells. *Science*.

[8] Lane A. N., Fan T. W. M. (2015). Regulation of Mammalian Nucleotide Metabolism and Biosynthesis. *Nucleic Acids Research*.

[9] Naushad S. M., Pavani A., Rupasree Y. (2012). Association of Aberrations in One-Carbon Metabolism With Molecular Phenotype and Grade of Breast Cancer. *Molecular Carcinogenesis*.

[10] Leek J. T., Johnson W. E., Parker H. S., Jaffe A. E., Storey J. D. (2012). The SVA Package for Removing Batch Effects and Other Unwanted Variation in High-Throughput Experiments. *Bioinformatics*.

[11] Darmanis S., Sloan S. A., Croote D. (2017). Single-Cell RNA-Seq Analysis of Infiltrating Neoplastic Cells at the Migrating Front of Human Glioblastoma. *Cell Reports*.

[12] Barkley D., Moncada R., Pour M. (2022). Cancer Cell States Recur Across Tumor Types and Form Specific Interactions With the Tumor Microenvironment. *Nature Genetics*.

[13] Aran D., Looney A. P., Liu L. (2019). Reference-Based Analysis of Lung Single-Cell Sequencing Reveals a Transitional Profibrotic Macrophage. *Nature Immunology*.

[14] Borcherding N., Voigt A. P., Liu V., Link B. K., Zhang W., Jabbari A. (2019). Single-Cell Profiling of Cutaneous T-Cell Lymphoma Reveals Underlying Heterogeneity Associated With Disease Progression. *Clinical Cancer Research*.

[15] Tirosh I., Izar B., Prakadan S. M. (2016). Dissecting the Multicellular Ecosystem of Metastatic Melanoma by Single-Cell RNA-seq. *Science*.

[16] Deng Y., Zheng Y., Li D. (2021). Expression Characteristics of Interferon-Stimulated Genes and Possible Regulatory Mechanisms in Lupus Patients Using Transcriptomics Analyses. *eBioMedicine*.

[17] Andreatta M., Carmona S. J. (2021). UCell: Robust and Scalable Single-Cell Gene Signature Scoring. *Computational and Structural Biotechnology Journal*.

[18] Zhang Y., Liu T., Hu X. (2021). CellCall: Integrating Paired Ligand-Receptor and Transcription Factor Activities for Cell-Cell Communication. *Nucleic Acids Research*.

[19] Wu Y., Yang S., Ma J. (2022). Spatiotemporal Immune Landscape of Colorectal Cancer Liver Metastasis at Single-Cell Level. *Cancer Discovery*.

[20] Cable D. M., Murray E., Zou L. S. (2022). Robust Decomposition of Cell Type Mixtures in Spatial Transcriptomics. *Nature Biotechnology*.

[21] Tanevski J., Flores R. O. R., Gabor A., Schapiro D., Saez-Rodriguez J. (2022). Explainable Multiview Framework for Dissecting Spatial Relationships From Highly Multiplexed Data. *Genome Biology*.

[22] Yoshihara K., Shahmoradgoli M., Martínez E. (2013). Inferring Tumour Purity and Stromal and Immune Cell Admixture From Expression Data. *Nature Communications*.

[23] Thorsson V., Gibbs D. L., Brown S. D. (2019). The Immune Landscape of Cancer. *Immunity*.

[24] Charoentong P., Finotello F., Angelova M. (2017). Pan-Cancer Immunogenomic Analyses Reveal Genotype-Immunophenotype Relationships and Predictors of Response to Checkpoint Blockade. *Cell Reports*.

[25] Liu J., Li S., Liang J. (2019). ITLNI Identified by Comprehensive Bioinformatic Analysis as a Hub Candidate Biological Target in Human Epithelial Ovarian Cancer. *Cancer Management and Research*.

[26] Low V., Li Z., Blenis J. (2022). Metabolite Activation of Tumorigenic Signaling Pathways in the Tumor Microenvironment. *Science Signaling*.

[27] Saveljeva S., Sewell G. W., Ramshorn K. (2022). A Purine Metabolic Checkpoint That Prevents Autoimmunity and Autoinflammation. *Cell Metabolism*.

[28] Alvarado A. G., Thiagarajan P. S., Mulkearns-Hubert E. E. (2017). Glioblastoma Cancer Stem Cells Evade Innate Immune Suppression of Self-Renewal Through Reduced TLR4 Expression. *Cell Stem Cell*.

[29] Linden J., Koch-Nolte F., Dahl G. (2019). Purine Release, Metabolism, and Signaling in the Inflammatory Response. *Annual Review of Immunology*.

[30] Kuroki L., Guntupalli S. R. (2020). Treatment of Epithelial Ovarian Cancer. *BMJ*.

[31] Bahreyni A., Samani S. S., Rahmani F. (2018). Role of Adenosine Signaling in the Pathogenesis of Breast Cancer. *Journal of Cellular Physiology*.

[32] Di Virgilio F., Adinolfi E. (2017). Extracellular Purines, Purinergic Receptors and Tumor Growth. *Oncogene*.

[33] Torre L. A., Trabert B., DeSantis C. E. (2018). Ovarian Cancer Statistics, 2018. *CA: A Cancer Journal for Clinicians*.

[34] Tanyi J. L., Chu C. S. (2012). Dendritic Cell-Based Tumor Vaccinations in Epithelial Ovarian Cancer: A Systematic Review. *Immunotherapy*.

[35] Nurmik M., Ullmann P., Rodriguez F., Haan S., Letellier E. (2020). In Search of Definitions: Cancer-Associated Fibroblasts and Their Markers. *International Journal of Cancer*.

[36] Saitoh M. (2018). Involvement of Partial EMT in Cancer Progression. *Journal of Biochemistry*.

[37] Zavadil J., Böttinger E. P. (2005). TGF-*β* and Epithelial-to-Mesenchymal Transitions. *Oncogene*.

[38] Roane B. M., Arend R. C., Birrer M. J. (2019). Review: Targeting the Transforming Growth Factor-Beta Pathway in Ovarian Cancer. *Cancers (Basel)*.

[39] Baulida J. (2017). Epithelial-to-Mesenchymal Transition Transcription Factors in Cancer-Associated Fibroblasts. *Molecular Oncology*.

[40] Lheureux S., Gourley C., Vergote I., Oza A. M. (2019). Epithelial Ovarian Cancer. *Lancet*.

[41] Morand S., Devanaboyina M., Staats H., Stanbery L., Nemunaitis J. (2021). Ovarian Cancer Immunotherapy and Personalized Medicine. *International Journal of Molecular Sciences*.

[42] Savas P., Virassamy B., Ye C. (2018). Single-Cell Profiling of Breast Cancer T Cells Reveals a Tissue-Resident Memory Subset Associated With Improved Prognosis. *Nature Medicine*.

[43] Schoutrop E., Moyano-Galceran L., Lheureux S. (2022). Molecular, Cellular and Systemic Aspects of Epithelial Ovarian Cancer and Its Tumor Microenvironment. *Seminars in Cancer Biology*.

[44] Liu J., Hong S., Yang J. (2022). Targeting Purine Metabolism in Ovarian Cancer. *Journal of Ovarian Research*.

[45] Gutiérrez-Melo N., Baumjohann D. (2023). T Follicular Helper Cells in Cancer. *Trends in Cancer*.

[46] Ukita M., Hamanishi J., Yoshitomi H. (2022). CXCL13-Producing CD4+ T Cells Accumulate in the Early Phase of Tertiary Lymphoid Structures in Ovarian Cancer. *JCI Insight*.

[47] Proust B., Radić M., Vidaček N. Š. (2021). NME6 Is a Phosphotransfer-Inactive, Monomeric NME/NDPK Family Member and Functions in Complexes at the Interface of Mitochondrial Inner Membrane and Matrix. *Cell & Bioscience*.

[48] Perina D., Bosnar M. H., Mikoč A., Müller W. E. G., Ćetković H. (2011). Characterization of Nme6-Like Gene/Protein From Marine Sponge Suberites domuncula. *Naunyn-Schmiedeberg’s Archives of Pharmacology*.

[49] El-Maarri O., Jamil M. A., Köster M. (2021). Stratifying Cumulus Cell Samples Based on Molecular Profiling to Help Resolve Biomarker Discrepancies and to Predict Oocyte Developmental Competence. *International Journal of Molecular Sciences*.

[50] Ke J., Lou J., Zhong R. (2016). Identification of a Potential Regulatory Variant for Colorectal Cancer Risk Mapping to 3p21.31 in Chinese Population. *Scientific Reports*.

[51] Edwards L., Gupta R., Filipp F. V. (2016). Hypermutation of DPYD Deregulates Pyrimidine Metabolism and Promotes Malignant Progression. *Molecular Cancer Research*.

