# Supplementary figures and images for: Single-Cell and Spatial Transcriptomics Explore Purine Metabolism–Related Prognostic Risk Model and Tumor Immune Microenvironment Modulation in Ovarian Cancer
Source: Hum Mutat. 2025 May 9;2025:5530325. doi: 10.1155/humu/5530325 (PMC12084792; doi:10.1155/humu/5530325)

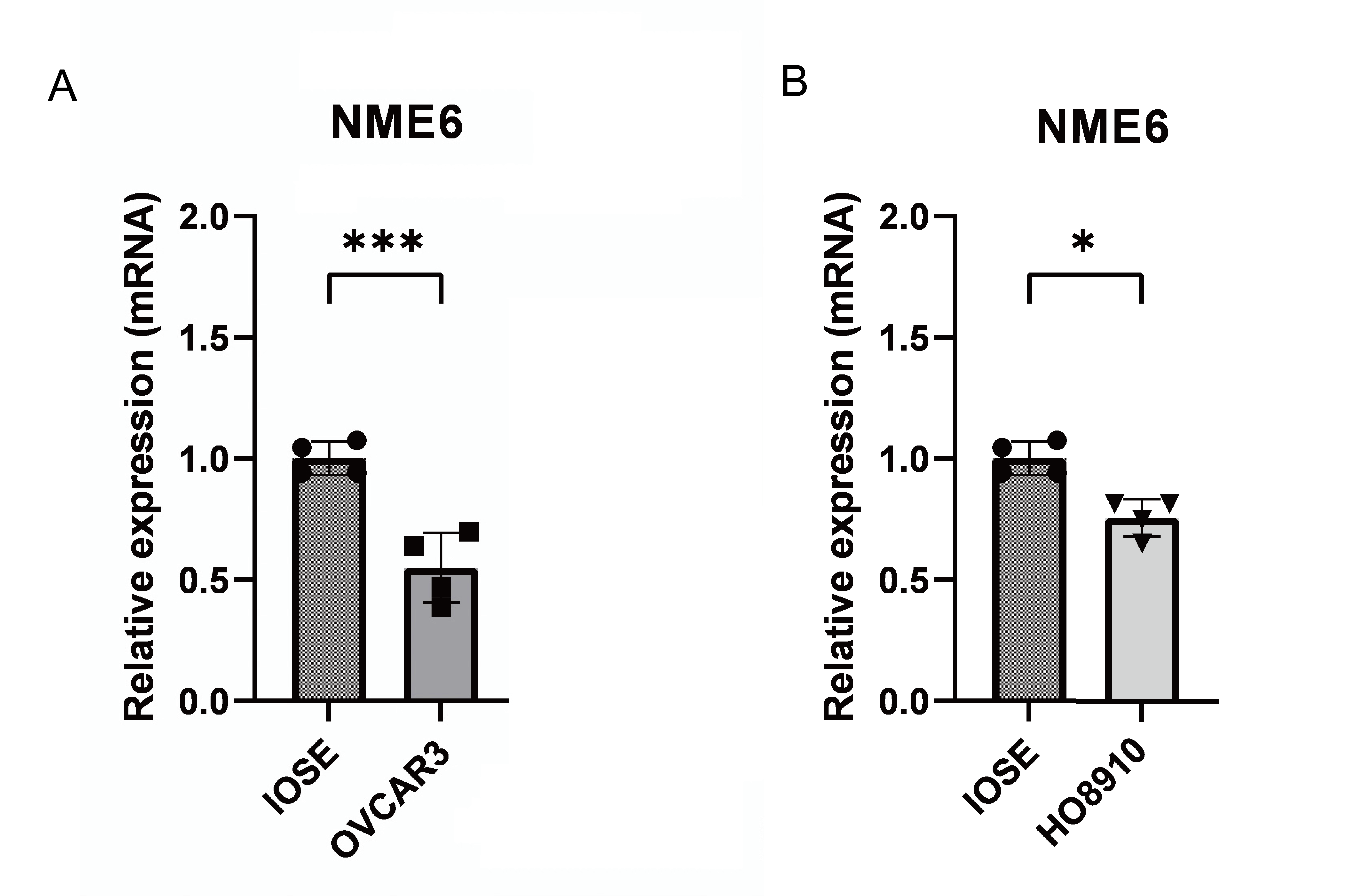

Supplement: Supporting Information 1 — Figure S1. The expression of NME6 in IOSE and OVCAR3 cell lines. [file 5530325.f1.png]
