# Supplementary material for: Single-Cell and Spatial Transcriptomics Explore Purine Metabolism–Related Prognostic Risk Model and Tumor Immune Microenvironment Modulation in Ovarian Cancer
Source: Hum Mutat. 2025 May 9;2025:5530325. doi: 10.1155/humu/5530325 (PMC12084792; doi:10.1155/humu/5530325)
Supplement: Supporting Information 6 — Table S5. Relative expression of NME6 in normal and tumor cell lines. [file 5530325.f6.docx]

|  | lOSE | OVCAR3 | HO8910 |
| --- | --- | --- | --- |
| 1 | 0.943 | 0.639 | 0.815 |
| 2 | 1.046 | 0.388 | 0.815 |
| 3 | 1.075 | 0.471 | 0.653 |
| 4 | 0.943 | 0.700 | 0.750 |

Supplementary Table 5 Relative expression of NME6 in normal and tumor cell lines
